# Left Ventricular Diastolic Dysfunction Predicts Global Longitudinal Strain Recovery after Surgical Aortic Valve Replacement

**DOI:** 10.3390/diagnostics14192176

**Published:** 2024-09-29

**Authors:** Francesca Bonanni, Sabina Caciolli, Martina Berteotti, Andrea Grasso Granchietti, Valentina Tozzetti, Noemi Cenni, Chiara Servoli, Marta Bandini, Enrico Marchi, Stefano Del Pace, Pierluigi Stefano, Niccolò Marchionni

**Affiliations:** 1 Division of General Cardiology, Department of Cardiac, Thoracic and Vascular Medicine, Azienda Ospedaliero-Universitaria Careggi, 50134 Florence, Italy; sabinacaciolli@gmail.com (S.C.); martina.berteotti@gmail.com (M.B.); andrea.grasso@unifi.it (A.G.G.); valentina.tozzetti@unifi.it (V.T.); noemi.cenni@unifi.it (N.C.); chiara.servoli@unifi.it (C.S.); marta.bandini@unifi.it (M.B.); enrico.marchi1@gmail.com (E.M.); delpaces@aou-careggi.toscana.it (S.D.P.); niccolo.marchionni@unifi.it (N.M.); 2 Health Science Interdisciplinary Center, Sant’Anna School of Advanced Studies, 56127 Pisa, Italy; 3 Department of Experimental and Clinical Medicine, University of Florence, 50134 Florence, Italy; 4 Division of Cardiac Surgery, Careggi University Hospital, 50134 Florence, Italy; pierluigi.stefano@unifi.it

**Keywords:** global longitudinal strain, aortic stenosis, cardiac surgery

## Abstract

Background and Objectives: In patients with severe aortic stenosis (AS), left ventricular systolic dysfunction is one of the main predictors of adverse events after surgical aortic valve replacement (SAVR). However, more patients undergo surgery earlier, often with preserved systolic function. In these cases, global longitudinal strain (GLS) has been proposed as a marker of ventricular remodeling post-surgery. This study aims to evaluate GLS variation in patients undergoing SAVR and explore differences across the diastolic dysfunction classes. Methods: From June 2020 to March 2023, patients with AS and preserved ejection fraction (EF) requiring SAVR were enrolled. Echocardiographic evaluations were conducted preoperatively, seven days post-surgery, and twelve months after surgery. Patients were divided into two groups based on the severity of diastolic dysfunction: Group A (grade I) and Group B (grades II–III). Results: The final analysis included 108 patients (mean age 71.3 ± 7.2 years). Twenty-two patients (20.4%) also underwent coronary artery bypass grafting (CABG). The preoperative EF averaged 61.6 ± 6.03%, with no significant differences between groups. Preoperative GLS was 16 ± 4.3%, decreasing to 12.8 ± 3.4% postoperatively (*p* < 0.0001). GLS was comparable between the groups preoperatively (*p* = 0.185) and postoperatively (0.854). After twelve months, GLS improved in both groups (Group A: 17.7 ± 3.4%, Group B: 15.7 ± 3.2%, *p* < 0.0001), but only Group A showed significant improvement from preoperative values (*p* = 0.018). SAVR improved GLS regardless of CABG intervention. Conclusions: SAVR in patients with preserved LVEF results in an early reduction in GLS, regardless of diastolic dysfunction. After twelve months, GLS improved significantly, with significant recovery only in patients with mild dysfunction.

## 1. Introduction

Aortic valve stenosis (AS) is the most prevalent acquired heart valve disease in Western countries [1], with a degenerative etiology; in a minority of cases, it is the consequence of rheumatic or infective endocarditis. The prevalence of AS rises with increasing age, affecting nearly 3% of individuals over the age of 75 years, and this valvular disease is characterized by the progressive narrowing of the aortic valve orifice, leading to increased resistance against left ventricular outflow [2,3].

Over time, such a significant pressure overload to the left ventricle (LV) triggers hypertrophic remodeling, which initially helps maintain cardiac output. AS is often associated with left ventricular diastolic dysfunction, while systolic dysfunction appears later during the natural course of the disease; some patients are severely symptomatic, manifesting dyspnea, angina, and syncope, and may have episodes of heart failure even in the presence of preserved LV systolic function [4]. However, defining cases as truly asymptomatic may be challenging, particularly in older and sedentary patients.

Severe, symptomatic AS has a poor prognosis [5], and timely aortic valve replacement (AVR) is mandatory when symptoms or LV systolic dysfunction occur in order to improve long-term prognosis [6]. Without AVR, the survival rate drops dramatically, with a mortality rate exceeding 50% within two years for those with symptomatic AS. Early identification of patients who will benefit from AVR is critical, particularly as the progression of AS may be difficult to predict reliably. Once symptoms manifest, it is often too late to prevent irreversible damage to the myocardium. Current guidelines emphasize early intervention, not only to relieve the mechanical obstruction but also to prevent the downstream effects of prolonged pressure overload on the myocardium, including fibrosis and heart failure. According to guidelines, patients are candidates for surgical (SAVR) or transcatheter (TAVR) AVR based on age, surgical risk, and technical feasibility [6].

LV systolic dysfunction is one of the main predictors of adverse events after AVR, increasing mortality and postoperative complications [7,8]. Subtle LV dysfunction may remain unrevealed by standard routine echocardiography in patients with preserved left ventricular ejection fraction (LVEF). Speckle-tracking echocardiography has been shown to be a promising tool for revealing even initial LV dysfunction [9]. In particular, global longitudinal strain (GLS) proved to be a robust prognostic marker in patients with severe AS and preserved LVEF [5], in whom reduced GLS is usually associated with myocardial fibrosis and ventricular remodeling [10,11,12]. Conversely, GLS has been shown to improve remarkably after AVR [5,13,14]. However, the correlation between GLS modifications and baseline LV diastolic dysfunction has not yet been investigated. Understanding this correlation may reveal information of crucial clinical relevance because diastolic dysfunction, which is frequently present in AS patients, may have a substantial impact on the prediction of the recovery of myocardial function post-AVR. This study aims to fill this gap in knowledge by exploring how preoperative diastolic function influences the improvement of GLS after SAVR. Exploring this issue might provide valuable insights into the potential benefits of early AVR before the development of advanced diastolic dysfunction, thereby improving long-term outcomes for AS patients

## 2. Materials and Methods

### 2.1. Study Population

This observational, prospective, monocentric study enrolled consecutive patients with severe AS (defined as an aortic valve area, AVA, <1.0 cm^2^) [6], with an indication for SAVR according to the most recent ESC guidelines [6], who were evaluated at the cardiac surgery department of our hospital from June 2020 to March 2023. The decision to perform SAVR was made after a thorough discussion by the heart team, which included cardiologists, cardiac surgeons, and anesthesiologists, ensuring that all aspects of the patient’s health, including their surgical risk and comorbidities, were considered. This interdisciplinary approach permits a balanced evaluation of whether surgical or transcatheter intervention is more appropriate for each patient. SAVR was preferred in patients without any contraindication to open-heart surgery and who were considered to be at an acceptable surgical risk.

Inclusion criteria were (1) severe, symptomatic, high-flow, high-gradient AS with an indication for SAVR, with or without associated coronary artery bypass graft surgery (CABG); (2) LVEF > 50%; (3) age > 18 years. The inclusion of patients undergoing CABG allowed for the investigation of potential confounding effects of coronary artery disease on recovery of myocardial function after SAVR. This was important because coronary artery disease and aortic stenosis often coexist, and addressing both conditions simultaneously could influence the postoperative recovery trajectory.

Exclusion criteria were (1) atrial fibrillation; (2) more than mild associated aortic valve regurgitation; (3) previous heart valve surgery; (4) sub-valvular stenosis or endocarditis; (5) concomitant mitral valve disease requiring surgery; and (6) poor acoustic window.

Clinical and instrumental data were collected immediately before surgery and in the early postoperative period (seven days after surgery) with clinical, electrocardiographic, and echocardiographic data recording. Furthermore, all patients were scheduled for a 12-month follow-up visit, when they were re-evaluated clinically and by echocardiography. The study was approved by the Ethics Committee of Careggi University Hospital, and informed consent for access to individual data was obtained from all patients.

### 2.2. Echocardiographic Evaluation

Comprehensive transthoracic 2DE and Doppler echocardiographic data were obtained by three experienced cardiologists (SC, MB, SDP) using a commercially available ultrasound system (Vivid 9, General Electric Healthcare, Chicago, IL, USA). The use of such high-end imaging technology allowed for precise measurement and quantification of key echocardiographic parameters, ensuring a high degree of accuracy and reproducibility across different operators. Additionally, the cardiologists’ expertise in the study ensured consistency in image acquisition and interpretation, which is essential when evaluating subtle changes in myocardial strain or diastolic parameters that might not be apparent in standard echocardiography. The data were stored and analyzed offline on a dedicated workstation (EchoPAC version 202, General Electric Healthcare, Chicago, IL, USA).

Patients were positioned in left lateral decubitus. Data were acquired in the parasternal (long- and short-axis views) and apical views (two-, three-, and four-chamber apical views).

LV thickness and diameters were measured from the parasternal long-axis view. LV mass was calculated for each exam using Devereux’s formula and indexed by body surface area; LV end-diastolic and end-systolic volumes were measured from the apical 2- and 4-chamber views. The LVEF was calculated using Simpson’s rule [15].

AS severity was assessed by calculating AVA from the continuity equation. The mean and peak transaortic pressure gradients were calculated by averaging the gradients during systole with the CW Doppler [16]. LV GLS was assessed with speckle-tracking analysis from the three standard 2D apical views. All images were recorded using high-frame-rate loops (50–80 Hz) for accurate software-based analysis. Mean GLS was determined by averaging all values of the 18 segments of the three views. The GLS values, usually reported as negative percentages, are expressed as absolute values in the present work.

LV diastolic function was fully assessed according to the recent updated guidelines [17]: tricuspid regurgitation velocity (TRV) was measured with continuous wave (CW) Doppler; left atrial volume indexed (LAVI) was calculated from apical views; values of early (E wave) and late (A wave) trans-mitral velocities, the E/A ratio, and the E-deceleration time were obtained from pulsed-wave (PW) Doppler recordings; the peak early diastolic velocity (septal and lateral e’) and the E/e’ ratio were obtained by tissue Doppler.

Following the most recent international guidelines [17], patients were classified by three levels of diastolic dysfunction (I–III), which were used to distinguish two groups: A for grade I (mild) and B for grade II–III (moderate/severe) diastolic dysfunction

### 2.3. Statistical Analysis

Categorical variables were expressed as absolute and percentage values and compared between groups with a chi-squared test. Continuous variables were expressed as mean ± standard deviation (SD) or median and interquartile range (IQR) in case of non-normal distribution and compared with Student’s *t*-test and non-parametric tests as appropriate. Analysis of variance was performed using the ANOVA test and non-parametric tests as appropriate. A *p* < 0.05 was considered statistically significant. Statistical analysis was performed with SPSS for Macintosh (v. 28.0).

## 3. Results

### 3.1. Baseline Characteristics

One hundred eight consecutive patients were enrolled in the study. Their mean age was 71.3 ± 7.2 years, and 57 were men (52.8%; Table 1).

Ischemic heart disease with an indication for CABG coexisted in 22 patients (20.4%), 6 of whom (27.3%) for acute coronary syndromes and 4 (18.2%) for three-vessel disease. Most patients had mild diastolic dysfunction (69, 63.9%, Group A; Table 1).

Notably, there was a significant difference in sex distribution between the two groups (*p* = 0.029). In Group A, 42 patients (60.9%) were men, whereas only 15 patients (38.5%) in Group B were men, indicating a higher proportion of women with moderate-to-severe diastolic dysfunction.

The mean BMI was significantly higher in Group B compared with Group A (28.08 ± 4.1 kg/m^2^ vs. 26.18 ± 3.7 kg/m^2^, *p* = 0.017), suggesting that patients with more severe diastolic dysfunction tended to have a higher BMI. The presence of comorbidities such as hypertension, diabetes, dyslipidemia, and smoking history did not significantly differ between the two groups. However, chronic kidney disease was more prevalent in Group B (15.4%) compared with Group A (4.3%), although this difference did not attain statistical significance (*p* = 0.069). The need for CABG during surgery was similar between the two groups, with 15 patients (21.7%) in Group A and 7 patients (17.9%) in Group B undergoing CABG in addition to aortic valve replacement (*p* = 0.804). Significant coronary artery disease of the left main coronary artery (LMCA) was observed in 4 patients—3 in Group A and 1 in Group B (*p* = 0.746)—while for the circumflex artery, the prevalence was 11 patients—8 in Group A and 3 in Group B (*p* = 0.647). For the left anterior descending artery (LAD), it was 14 patients: 9 in Group A and 5 in Group B (*p* = 0.604). For the right coronary artery (RCA), 13 patients were affected: 8 in Group A and 5 in Group B (*p* = 0.421).

Hospital stay was also slightly longer in Group B, with a median of 12 days compared with 10 days in Group A, though this difference was not statistically significant (*p* = 0.187).

The echocardiographic parameters of our study population are summarized in Table 2.

The LV mass indexed to body surface area (LV mass/BSA) was slightly higher in Group B preoperatively (134 ± 29.6 g/m^2^) compared with Group A (127.8 ± 30.2 g/m^2^), though the difference was not statistically significant (*p* = 0.238). In the early postoperative period, both groups showed a significant reduction in LV mass (*p* < 0.001 for both). In terms of left atrial volume index (LAVI), Group B had significantly higher values preoperatively (44.8 ± 12.4 mL/m^2^ vs. 35.9 ± 12.1 mL/m^2^, *p* < 0.001), and this difference persisted postoperatively (40.9 ± 13.2 mL/m^2^ vs. 30.9 ± 11.5 mL/m^2^, *p* < 0.001). Both groups showed a significant reduction in LAVI postoperatively (*p* < 0.001 for both groups). Systolic pulmonary artery pressure (PAPS) was significantly higher in Group B preoperatively (33 mmHg vs. 28.5 mmHg, *p* = 0.001). Postoperatively, both groups showed a reduction in PAPS (Group A: 26 mmHg, Group B: 28 mmHg, *p* = 0.071), though the reduction was statistically significant only for Group B (*p* = 0.010) but not for Group A (*p* = 0.0263).

The peak aortic valve gradient was significantly higher in Group B preoperatively (95.3 ± 28.8 mmHg vs. 80.7 ± 26.2 mmHg, *p* = 0.010). Both groups experienced a significant reduction in peak gradient postoperatively (*p* < 0.001 for both), with no significant difference between groups in the early postoperative period (*p* = 0.630).

The aortic valve area (AVA) was 0.73 ± 0.13 cm^2^ in Group A and 0.65 ± 0.13 cm^2^ in Group B (*p* = 0.001). The indexed aortic valve area (iAVA) was 0.40 ± 0.09 cm^2^/m^2^ in Group A and 0.37 ± 0.09 cm^2^/m^2^ in Group B (*p* = 0.045).

The prevalence of mild mitral regurgitation was 23 cases (21.3%): 13 in Group A and 10 in Group B (*p* = 0.407). The prevalence of mild aortic regurgitation was 31 cases (28.7%): 20 in Group A and 11 in Group B (*p* = 0.931).

The mean GLS values before surgery were 16 ± 4.3%; at early postoperative examination, we observed a reduction to 12.8 ± 3.4%, *p* < 0.0001. Mean GLS was similar between patients in the two classes of diastolic dysfunction at both preoperative (*p* = 0.185) and early postoperative (*p* = 0.854) examination (Table 2).

The analysis was repeated in the 54 and 32 patients undergoing SAVR without CABG in groups A and B to verify whether coronary revascularization might be a confounder. Mean GLS was 16.6 ± 3.8% before surgery and 13.2 ± 2.9% in the early postoperative evaluation, *p* < 0.0001. Also in this case, values were similar in the two diastolic dysfunction groups: 16.5 ± 3.8% before surgery in Group A and 16.8 ± 4% in Group B (*p* = 0.718); 13.3 ± 2.8% in the early postoperative in Group A and 13 ± 3% in Group B (*p* = 0.778).

### 3.2. Follow-Up

Of the 108 patients included in the study, 86 (80%) completed a follow-up visit, whereas 2 (2%) died from non-cardiological causes, and 20 (18%) refused further cardiological examinations due to geographical distance.

At follow-up examination, no patient had signs or symptoms of heart failure. No patient had been re-hospitalized for heart failure, myocardial infarction, ventricular arrhythmias, or other cardiovascular causes over the follow-up after surgery, while three had had an episode of pneumonia, in one case requiring hospitalization.

A comprehensive echocardiographic examination was obtained in all patients participating in follow-up visits (Table 3).

The median LVEF was marginally but significantly greater, and LV mass was lower, in Group A. Group A exhibited a lower LV mass compared with Group B (90.2 ± 26.1 g/m^2^ vs. 103.4 ± 20.1 g/m^2^, *p* = 0.026), and both groups showed significant reductions in LV mass compared with baseline (*p* < 0.001 for both groups). The mean transvalvular gradient at follow-up was similarly low in the two groups (*p* = 0.216). From baseline to follow-up, LAVI was significantly reduced only in Group B (*p* = 0.018). Although Group B had higher LAVI values compared with Group A at follow-up, this difference was not statistically significant (38.3 ± 11.5 mL/m^2^ vs. 33.4 ± 12.0 mL/m^2^, *p* = 0.077).

**Figure 1:**
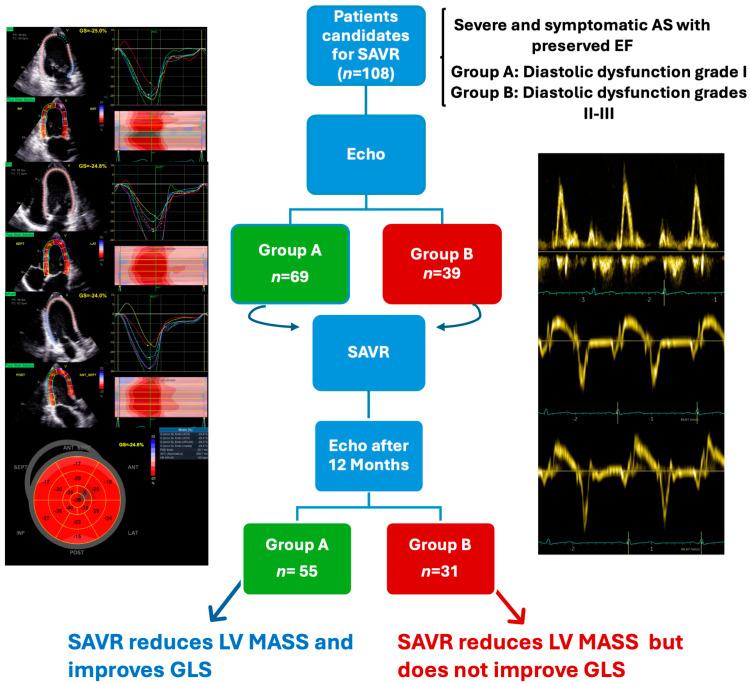
Central illustration: SAVR: surgical aortic valve replacement; LV: left ventricle; GLS: global longitudinal strain. Group A or A: diastolic dysfunction grade I; Group B or B: Diastolic dysfunction grades II–III.

Compared with the early postoperative values, 12-month GLS improved significantly in both groups (Table 3 and Figure 1).

**Figure 2:**
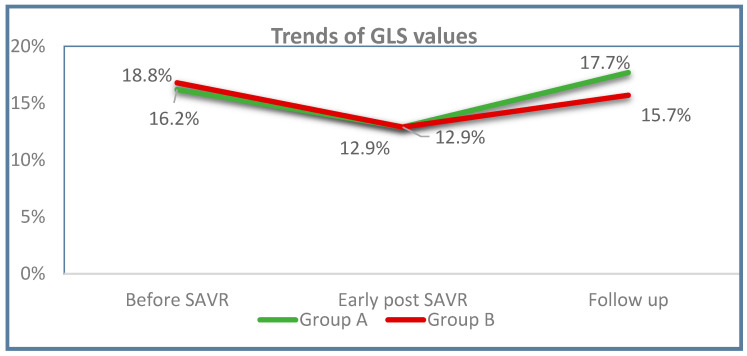
Trends of GLS values. SAVR: surgical aortic valve replacement; GLS: global longitudinal strain. Group A (green): diastolic dysfunction grade I; Group B (red): diastolic dysfunction grades II–III.

However, the improvement was significantly more considerable in the Group A patients with mild diastolic dysfunction (*p* = 0.018), in whom 12-month GLS increased over the baseline (Figure 2). The same analysis was repeated in patients undergoing isolated SAVR (without CABG). The mean GLS values were still significantly higher in Group A (18 ± 3.1%) than in B (16.3 ± 3.2%; *p* = 0.039) and, again, were improved from baseline in Group A only (*p* = 0.045).

## 4. Discussion

In the present study of patients with severe AS and preserved LVEF, the GLS before SAVR was reduced in most patients and, after an acute decrease in the early postoperative period, improved over the baseline only in patients with mild baseline diastolic dysfunction at 1 year follow-up.

Patients with severe AS and preserved LV function often present a decreased GLS, which suggests subclinical LV systolic dysfunction despite normal LVEF. In fact, the reduction in GLS usually precedes symptom onset [18,19,20], and it has been associated with a less favorable prognosis [21]. The ability of GLS to detect subclinical myocardial damage before overt symptoms develop underscores its clinical utility. Incorporating GLS into routine diagnostic protocols may allow for more accurate risk stratification, especially in patients who may appear asymptomatic but are already experiencing detrimental cardiac changes.

In some studies, a GLS < 15% or 16% has been taken to indicate initial LV dysfunction, but an optimal cut-off remains to be identified [18,22,23,24]. Determining an appropriate GLS threshold will be crucial for clinical decision making.

Several studies have demonstrated that SAVR improves GLS, especially in patients with elevated transvalvular gradients [5,14]. Indeed, SAVR causes a dramatic decline in LV pressure overload, leading to reduced wall stress and, over time, reverse remodeling with improved transmural myocardial perfusion and, finally, enhanced LV systolic performance. The timing of intervention remains critical, as earlier relief of pressure overload may enhance the potential for myocardial recovery. Notably, a persistently reduced GLS one year after SAVR has been associated with a less favorable long-term prognosis [20]. In this setting, it would be relevant to identify those patients who may benefit the most from surgery. Persistent GLS reductions suggest ongoing myocardial damage or incomplete recovery. These individuals may require closer postoperative follow-up and possibly adjunctive medical therapies aimed at promoting further reverse remodeling and improving myocardial strain. Targeting these patients with personalized therapeutic strategies could enhance their long-term outcomes and mitigate the risk of adverse events.

The present study found that patients with more severe baseline diastolic dysfunction did not significantly improve GLS at follow-up. Diastolic dysfunction is present in virtually all patients with AS and can affect their outcomes [25]. Some small retrospective studies have already shown that patients with more severe diastolic dysfunction have a greater degree of remodeling and worse outcomes after AVR [26,27]. It can be hypothesized that a higher degree of LV remodeling might hamper GLS improvement or require more time to achieve it.

Beyond GLS, in patients with mild diastolic dysfunction, we also observed that LV mass was reduced to a greater extent after SAVR, confirming a more pronounced reverse remodeling in this group. This supports the concept that early-stage diastolic dysfunction represents a more favorable substrate for recovery following SAVR. The significant reduction in LV mass in these patients indicates that hypertrophy remains relatively plastic and responsive to changes in hemodynamics when the intervention occurs early enough.

It is important to note that the mean values of GLS were reduced to about 12% in the immediate postoperative period, without significant differences in patients with different diastolic dysfunctions. Early postoperative reductions in GLS are likely multifactorial and influenced by the immediate effects of surgery, such as myocardial stunning, altered loading conditions, and inflammatory responses [28,29]. Recognizing that this decline is transient is essential for interpreting early postoperative echocardiographic findings.

Of relevance, the same results were confirmed in patients undergoing isolated SAVR, excluding the coronary revascularization effect on GLS improvement. This consistency across patient groups suggests that the improvement in GLS is primarily related to the correction of AS rather than the concurrent treatment of coronary artery disease. Thus, isolated SAVR has a clear and direct benefit on myocardial strain.

Our study highlights the correlation between diastolic dysfunction and GLS and identifies diastolic dysfunction as a predictor of GLS recovery post-SAVR.

Therefore, we support introducing LV strain as a further indication for surgery in patients with severe AS [30]. Similarly, we claim that diastolic dysfunction should also be considered when deciding the indication for valve replacement before LV systolic dysfunction or overt symptoms occur [18,20]. In fact, we found that SAVR, when patients have not yet developed a marked diastolic dysfunction, is associated with a more extensive—and perhaps an earlier—reverse remodeling, as suggested by higher GLS values at follow-up. This further reinforces the notion that surgical intervention should not be delayed until the later stages of diastolic dysfunction. Instead, early identification of impaired LV strain and mild diastolic dysfunction could serve as key factors for surgery, potentially leading to more favorable long-term outcomes.

The present study has some limitations that need to be acknowledged. First, the study sample was relatively small, with an even smaller number of patients with moderate-to-severe diastolic dysfunction. Moreover, our primary study outcome was represented by changes in GLS at follow-up, while we could not find any association with one-year adverse events, an outcome for which our study was vastly underpowered, also because of the limited follow-up duration.

## 5. Conclusions

In our study, patients with severe AS and mild diastolic dysfunction undergoing SAVR showed a remarkable improvement in GLS after twelve months, regardless of CABG intervention. The same did not occur in those with moderate-to-severe diastolic dysfunction. Further studies with larger populations and longer follow-ups are required to confirm these findings and explore the association between diastolic dysfunction, GLS recovery, and clinical outcomes.

## Figures and Tables

**Table 1:** Baseline characteristics of our study population at preoperative evaluation according to diastolic dysfunction groups.

Variables	Total (*n* = 108)	Group A (*n* = 69)	Group B (*n* = 39)	*p*-Value
Sex (male), *n* (%)	57 (52.8)	42 (60.9)	15 (38.5)	0.029
Age, (years), mean ± SD	71.3 ± 7.2	70.5 ± 7.6	72.6 ± 6.3	0.147
BMI (kg/m^2^), mean ± SD	26.87 ± 4	26.18 ± 3.7	28.08 ± 4.1	0.017
Hypertension, *n* (%)	76 (70.4)	46 (66.7)	30 (76.9)	0.283
Diabetes mellitus, *n* (%)	24 (22.2)	14 (20.3)	10 (25.6)	0.631
Dyslipidemia, *n* (%)	67 (62)	43 (62.3)	24 (61.5)	1.000
Chronic kidney disease, *n* (%)	9 (8.3)	3 (4.3)	6 (15.4)	0.069
Smoke, *n* (%)	51 (47.2)	28 (40.6)	23 (59)	0.074
Indication to CABG, *n* (%)	22 (20.4)	15 (21.7)	7 (17.9)	0.804
Euroscore II (%), median [IQR]	1.43 [1–2.2]	1.44 [1–2.2]	1.43 [1–2.2]	1.000
Hospital stay (days), median [IQR]	11 [8–14.8]	10 [8–13.5]	12 [10–17]	0.187

Continuous variables are presented as median [IQR] or mean ± SD, whereas categorical variables are presented as *n* (%). Group A: diastolic dysfunction grade I; Group B: diastolic dysfunction grades II–III. BMI: body mass index; CABG: coronary artery bypass graft surgery.

**Table 2:** Echocardiographic parameters.

	Preoperative				Early Postoperative						
Variables	Total (*n* = 108)	Group A (*n* = 69)	Group B (*n* = 39)	*p* A1 vs. B1	Total (*n* = 108)	Group A (*n* = 6)	Group B (*n* = 39)	*p* A2 vs. B2	*p* Total 1 vs. 2	*p* A1 vs. A2	*p* B1 vs. B2
LVEF (%)	63 [58–65]	61 [57.5–65]	64 [60–66]	0.562	59 [56–61]	60 [56–60]	60 [57–63]	0.721	<0.001	<0.001	0.002
LV/mass/BSA (g/m^2^)	130.4 ± 30.1	127.8 ± 30.2	134 ± 29.6	0.238	116.6 ± 35.6	111.9 ± 38.3	124.7 ± 29.2	0.074	<0.001	<0.001	0.027
IVS (mm)	13 [12–14]	13 [12–14]	13 [12–14]	0.338	12 [11–13]	12 [11–13]	13 [11–13]	0.292	<0.001	0.032	0.010
PWT (mm)	11 [11–12]	11 [10.25–12]	12 [11–12]	0.010	11 [10–12]	11 [10–12]	11 [10–12]	0.478	0.320	0.683	0.022
LVD (mm)	50 [45–53]	50 [45.5–54]	48 [45–52]	0.253	47 [45–53]	47 [45–52]	48 [45–54]	0.592	0.04	<0.001	0.837
LAVI (mL/m^2^)	39.1 ± 12.9	35.9 ± 12.1	44.8 ± 12.4	<0.001	34.4 ± 13	30.9 ± 11.5	40.9 ± 13.2	<0.001	<0.001	0.001	0.036
TAPSE (mm)	22.5 ± 3.6	22.3 ± 3.5	23 ± 3.8	0.310	16 ± 3.1	16.6 ± 3.1	16.5 ± 3.3	0.905	<0.001	<0.001	<0.001
PAPS (mmHg)	29 [25–34]	28.5 [24–32.8]	33 [28–36]	0.001	26 [22–33]	26 [22–31]	28 [25–35]	0.071	0.009	0.263	0.010
RVD2 (mm)	28.4 ± 6.7	28.8 ± 6.6	27.9 ± 6.9	0.530	28.5 ± 5.9	28.8 ± 5.8	27.9 ± 6.2	0.435	0.975	0.988	0.966
Grad-Peak (mmHg)	86 ± 27.9	80.7 ± 26.2	95.3 ± 28.8	0.010	13.3 ± 4.3	13.0 ± 3.9	13.7 ± 4.8	0.630	<0.001	<0.001	<0.001
Grad-Mean (mmHg)	56.7 ± 19.1	54.11 ± 18.1	61.2 ± 20.1	0.065	9.2 ± 3.1	9.4 ± 3.15	9 ± 3.8	0.435	<0.001	<0.001	<0.001
Mean GLS (%)	16 ± 4.3	15.6 ± 4.3	16.7 ± 4.2	0.185	12.8 ± 3.4	12.7 ± 3.3	12.8 ± 3.5	0.854	<0.001	<0.001	<0.001

Continuous variables are presented as median [IQR] or mean ± SD. Group A or A: diastolic dysfunction grade I; Group B or B: diastolic dysfunction grades II–III; 1: preoperative; 2: early postoperative. LVEF: left ventricular ejection fraction; IVS: interventricular septum; PWT: posterior wall thickness; LVD: left ventricular diameter; LAVI: left atrial volume index; TAPSE: tricuspid annular plane systolic excursion; PAPS: systolic pulmonary artery pressure; RVD2: mid-cavity diameter of the right ventricle; Grad-Peak: aortic valve peak gradient; Grad-Mean: aortic valve mean gradient.

**Table 3:** Echocardiographic parameters at follow-up according to diastolic dysfunction groups.

Variables	Total (*n* = 86)	Group A (*n* = 55)	Group B (*n* = 31)	*p* A3 vs. B3	*p* Total 1 vs. 3	*p* A1 vs. A3	*p* B1 vs. B3
EF (%)	60 [59–65]	60 [60–65]	60 [58–63]	0.034	0.050	0.478	0.025
LV/mass/BSA (g)	95.1 ± 25.7	90.2 ± 26.1	103.4 ± 20.1	0.026	<0.001	<0.001	<0.001
IVS (mm)	11 [11,12]	11 [10–12]	12 [11–13]	0.050	<0.001	<0.001	0.003
PWT (mm)	10 [10–11]	10 [9–11]	11 [10–11]	0.010	<0.001	<0.001	0.827
LVD (mm)	45 [43–48]	45 [43–48]	45 [42–50]	0.825	<0.001	<0.001	0.016
LAVI (mL/m^2^)	35.2 ± 12.0	33.4 ± 12.0	38.3 ± 11.5	0.077	0.021	0.289	0.018
TAPSE (mm)	20.1 ± 3.2	19.7 ± 2.8	20.6 ± 3.7	0.208	<0.001	<0.001	0.003
PAPS (mmHg)	25 [20–29]	24 [20–26]	27 [22–33]	0.036	<0.001	0.004	0.031
RVD2 (mm)	30.0 ± 5.3	30.9 ± 5.0	28.7 ± 5.7	0.080	0.011	0.012	0.412
Grad-Peak (mmHg)	20.1 ± 7.4	19.3 ± 6.1	21.5 ± 8.9	0.198	<0.001	<0.001	<0.001
Grad-Mean (mmHg)	11.3 ± 4.5	10.8 ± 3.8	11.0 ± 5.5	0.216	<0.001	<0.001	<0.001
Mean GLS (%)	17.0 ± 3.4	17.7 ± 3.4	15.7 ± 3.2	0.011	0.087	0.018	0.221

Continuous variables are presented as median [IQR] or mean ± SD. Group A or A: diastolic dysfunction grade I; Group B or B: diastolic dysfunction grades II–III; 1: preoperative; 3: follow-up. IVS: interventricular septum; PWT: posterior wall thickness; LVD: left ventricular diameter; LAVI: left atrial volume index; TAPSE: tricuspid annular plane systolic excursion; PAPS: systolic pulmonary artery pressure; RVD2: mid-cavity diameter of the right ventricle; Grad-Peak: aortic valve peak gradient; Grad-Mean: aortic valve mean gradient; GLS: global longitudinal strain.

## Data Availability

Data are contained within the article.
